# The development and acceptability of an educational and training intervention for recruiters to neonatal trials: the TRAIN project

**DOI:** 10.1186/s12874-023-02086-1

**Published:** 2023-11-11

**Authors:** V. Smith, H. Delaney, A. Hunter, D. Torgerson, S. Treweek, C. Gamble, N. Mills, K. Stanbury, E. Dempsey, M. Daly, J. O’Shea, K. Weatherup, S. Deshpande, M. A. Ryan, J. Lowe, G. Black, D. Devane

**Affiliations:** 1 School of Nursing and Midwifery, University of Dublin, Trinity College Dublin, Dublin, Ireland; 2 Health Research Board-Trials Methodology Research Network (HRB-TMRN), University of Galway, Galway, Ireland; 3 School of Nursing and Midwifery, University of Galway, Galway, Ireland; 4 York Trials Unit, University of York, York, YO10 5DD UK; 5 Health Services Research Unit, Trial Forge, University of Aberdeen, Aberdeen, UK; 6 Liverpool Clinical Trials Centre, University of Liverpool, Liverpool, UK; 7 QuinteT, Population Health Sciences, Bristol Medical School, University of Bristol, Bristol, UK; 8 National Perinatal Epidemiology Unit (NPEU), Nuffield Department of Population Health, University of Oxford, Oxford, UK; 9 INFANT Centre, University College Cork, Cork, Ireland; 10 Irish Neonatal Health Alliance, Public and Patient Involvement Contributor, Bray, Co-Wicklow Ireland; 11 Public and Patient Involvement Contributor, Royal Hospital for Children, Glasgow, UK; 12 Public and Patient Involvement Contributor, Oxford, UK; 13 Princess Royal Hospital, Telford, UK; 14 Centre for Trials Research, College of Biomedical and Life Sciences, Cardiff University, Cardiff, UK; 15 Royal Hospital for Children and Young People, Edinburgh, UK

**Keywords:** Trial recruitment, Training recruiters, Intervention development, Neonatal trials

## Abstract

**Background:**

Suboptimal or slow recruitment affects 30–50% of trials. Education and training of trial recruiters has been identified as one strategy for potentially boosting recruitment to randomised controlled trials (hereafter referred to as trials). The **T**raining t**R**ial recruiters, **A**n educational **IN**tervention (TRAIN) project was established to develop and assess the acceptability of an education and training intervention for recruiters to neonatal trials. In this paper, we report the development and acceptability of TRAIN.

**Methods:**

TRAIN involved three sequential phases, with each phase contributing information to the subsequent phase(s). These phases were 1) evidence synthesis (systematic review of the effectiveness of training interventions and a content analysis of the format, content, and delivery of identified interventions), 2) intervention development using a Partnership (co-design/co-creation) approach, and 3) intervention acceptability assessments with recruiters to neonatal trials.

**Results:**

TRAIN, accompanied by a comprehensive intervention manual, has been designed for online or in-person delivery. TRAIN can be offered to recruiters before trial recruitment begins or as refresher sessions during a trial. The intervention consists of five core learning outcomes which are addressed across three core training units. These units are the trial protocol (Unit 1, 50 min, trial-specific), understanding randomisation (Unit 2, 5 min, trial-generic) and approaching and engaging with parents (Unit 3, 70 min, trial-generic). Eleven recruiters to neonatal trials registered to attend the acceptability assessment training workshops, although only four took part. All four positively valued the training Units and resources for increasing recruiter preparedness, knowledge, and confidence. More flexibility in how the training is facilitated, however, was noted (e.g., training divided across two workshops of shorter duration). Units 2 and 3 were considered beneficial to incorporate into Good Clinical Practice Training or as part of induction training for new staff joining neonatal units.

**Conclusion:**

TRAIN offers a comprehensive co-produced training and education intervention for recruiters to neonatal trials. TRAIN was deemed acceptable, with minor modification, to neonatal trial recruiters. The small number of recruiters taking part in the acceptability assessment is a limitation. Scale-up of TRAIN with formal piloting and testing for effectiveness in a large cluster randomised trial is required.

**Supplementary Information:**

The online version contains supplementary material available at 10.1186/s12874-023-02086-1.

## Background

Clinical decision-makers and policy and guideline developers often use the results of systematic reviews of randomised trials and other studies, to guide and inform healthcare practices. Randomised trials have long been considered the gold standard for testing the effectiveness of interventions, yet they are often wrought with challenges. One challenge is that of slow or suboptimal recruitment, with reports suggesting that about half of all trials do not meet their recruitment target or do so only with an extension to the original trial duration [1, 2]. For example, of 73 trials funded by the United Kingdom (UK) Medical Research Council (MRC) and the Health Technology Assessment (HTA) programme between 2002 and 2008, 55% of trials recruited to their target sample size and nearly half (45%) received an extension [3]. Similar issues have been reported in the United States. A study investigating the prevalence and associated economic impact of low enrolling clinical studies at a single academic medical centre found that of the 837 clinical studies terminated during the study period, 31.1% were closed because of low recruitment and at a cost of almost $1 million [4].

Under-recruiting or stopping a trial early due to poor recruitment has major implications for the study outcomes, not least a reduction in the study's statistical power [5]. Underpowering a trial adds uncertainty; for example, an underpowered study may report no difference between groups on clinically important outcomes when, in fact, a difference may exist. Other implications of poor recruitment or stopping a trial early include increased burden and resource waste, ethical issues, and reduced impact on clinical care as the research question may be insufficiently addressed to inform clinical practice [1, 6].

To boost recruitment or address slow recruitment, trial coordinators often engage in responsive activities, for example, altered or increased communication strategies [7], incentives [6], or formal site visits by the principal investigator [5], yet sufficient, robust evidence on the effectiveness of many of these activities is lacking. Uncertainties around trial elements that might potentially impact recruitment have also been explored in survey research [8–10]. To further explore and, importantly, prioritise trial recruitment uncertainties, the Health Research Board Trials Methodology Research Network (HRB-TMRN), in 2016, undertook the PRioRiTy study [11]. Using a James Lind Alliance-Priority Setting Partnership (JLA-PSP) approach, the PRioRiTy study identified and ranked the Top 10 priority questions for trial recruitment uncertainties. One thematic area that emerged in PRioRiTy was educating and training trial recruiters. This finding also reflects a ranking exercise involving members of UK Clinical Trial Units (CTUs), where training site staff was identified as the number one priority for future evaluative research [12].

Training recruiters has been found to improve enthusiasm for trials and build recruiter confidence in communicating about trials with patients [13]. Yet, evidence of the effectiveness of trial recruiter education and training interventions, and the types of training required, appears lacking [14]. For this reason, the **T**raining t**R**ial recruiters; **A**n educational **IN**tervention (TRAIN) project was established to develop and assess the acceptability of an education and training intervention for recruiters to neonatal trials. Acknowledging that all trials can experience recruitment challenges, we specifically chose neonatal trials as the focus for TRAIN because recruitment challenges to these trials can be compounded further by having to approach parents at a challenging time, that is, in the context of parental fear, worry and concern for a new baby who may be very unwell, and within a time scale that is often limited when making a decision [15].

## Methods

### Aim

**Fig. 1:**
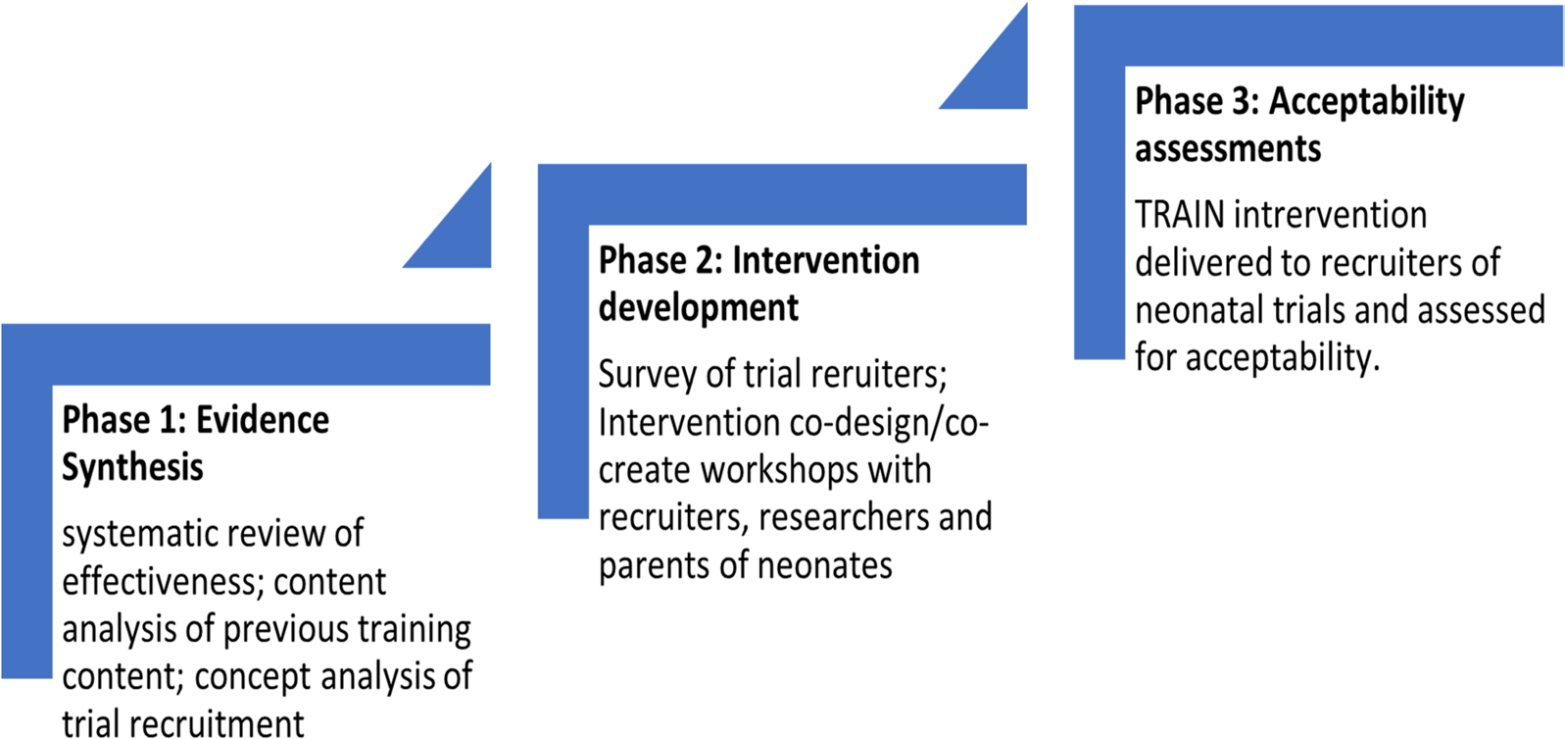
TRAIN project phases

We aimed to develop and assess the acceptability of an education and training intervention for recruiters to neonatal trials. The study involved three sequential phases to achieve this aim, with each phase contributing information directly to the subsequent phase(s). These phases were 1) evidence synthesis, 2) intervention development and 3) intervention acceptability assessment (Fig. 1).

### Phase 1: Evidence synthesis

The purpose of phase 1 was to gain preliminary information that could be used to inform aspects of phase 2. The evidence synthesis activity involved a systematic review of the effectiveness of existing training interventions, a content analysis of the content, format and delivery of existing training interventions, and a concept analysis of the concept ‘*trial recruitment’*. The findings of phase 1 have been reported elsewhere [14, 16, 17]; thus, in this paper, we report the conduct and findings of phases 2 and 3.

### Phase 2: Online survey of recruiters to neonatal trials

To ascertain the opinions of recruiters to neonatal trials on the specific education and training requirements that they believed would enhance trial recruitment and to provide data from the perspectives of ‘recruiter’ stakeholders to further inform the development of TRAIN, an online survey of recruiters to neonatal trials was conducted. The survey was designed using the findings from phase 1, especially the findings of the content analysis which provided information on recruiters’ preferences around training delivery format, training materials, duration of the training, and training content [17]. The draft survey was subjected to validity assessments by a panel of five expert trial recruiters, after which minor refinements were made. The final survey (Additional file 1) included a mix of multiple-choice, Likert scale and open-ended questions with free text boxes for comments.

The target population for the survey was all individuals involved in recruitment to neonatal trials, either directly or indirectly (i.e., in designing recruitment processes) across Ireland and the UK. As the number in the target population was unknown, we were unable to determine a study sample size estimate; however, our intention was to gain as many responses as possible. The survey was distributed online using the QuestionPro platform in November–December 2020 for four weeks, with a reminder sent at the end of week two. A purposive sampling approach was used, supplemented by snowball sampling, whereby the survey was advertised by email and on social media via neonatal trial networks, clinical trial units, neonatal trial research facilities, and at a neonatal research symposium (Nov 2020, Ireland), with a request to forward the survey to known others who were involved in recruitment to neonatal trials. Survey distribution was also supported by the National Perinatal Epidemiology Unit (NPEU) Clinical Trials Unit, the Irish Centre for Maternal and Child Health Research (INFANT) and the HRB-TMRN.

### Phase 2: Intervention development co-design/co-production workshops

The final stage of developing TRAIN involved Partnership methodology using co-design/co-production methods. The TRAIN Cooperative Intervention Development Committee (TCIDC) was established and met with members of the core research team in two arranged workshops to draft TRAIN. TCIDC members were purposively selected based on their expertise. Members were two neonatal clinicians, two neonatal research nurses, one neonatal trial manager and four Public and Patient Involvement (PPI) contributors (parents of neonates previously involved in a neonatal trial) from the UK and Ireland. The TRAIN research team initially proposed a draft of TRAIN based on the evidence syntheses and the online survey findings. This draft was shared with the TCIDC for review before the first workshop. The intervention development workshops took place in March and April 2021 (online via Zoom due to COVID restrictions). The workshops were of 2-h duration and, with informed consent, were recorded and transcribed (for memory and recall purposes only).

### Phase 3: Acceptability of TRAIN

In phase 3, TRAIN was delivered to neonatal trial recruiters to assess for acceptability and to gain their feedback. The target sample included all individuals who had ever been, or would be in the future, involved in recruiting to a neonatal trial. An invite to participate in the acceptability workshops was issued during October–November 2021 to international neonatal trial recruiters by email and social media, via neonatal trials networks, clinical trial units and neonatal trial research facilities, and via the NPEU Clinical Trials Unit, the INFANT Centre, and the HRB-TMRN. The invite included a link to register for one of three training dates in November 2021 which involved attending a 2-h online TRAIN intervention workshop and completing a five-minute before-and-after survey. Once participants registered for a training date, they were sent a confirmation email with a link to the online training and the baseline survey.

The education and training sessions were delivered via Zoom, and each training session was facilitated by two members of the TRAIN core research team (AH, VS, HD). As the participants were involved in recruiting to different trials, template content that outlined what would be presented in Unit 1 for specific trials was presented, rather than actual content. Examples of training resources were also shared with participants, thus allowing them to provide feedback on the content of Unit 1. Units 2 and 3 were presented as they would be in a real training scenario. At the end of each Unit, participants had an opportunity to share comments or feedback on the Unit and were reminded that further comments could be shared in the online follow-up survey.

The pre-and post-session surveys were designed to capture three outcome measures. These were recruiters’ perceived preparedness and self-confidence as neonatal trial recruiters, recruiters perceived rating of their knowledge of the trial information, and recruiters perceived satisfaction with the training intervention. The pre-and-post surveys (Additional file 2) included a mix of multiple-choice, Likert scale, and open-ended questions with free text boxes for participants to include any other information.

### Ethical conduct

Ethical approval for the study was granted by the School of Nursing and Midwifery, Trinity College Dublin, Research Ethics Committee, Ref: 14^th^ May 2019 (see *ethical approval and consent to participate* section for full details). The study was ethically conducted in accordance with the General Data Protection Regulations (GDPR) (2018) (https://gdpr-info.eu/).

## Results

### Online survey of recruiters to neonatal trials

Ninety-three recruiters responded to the survey representing clinicians involved in front line recruitment (*n* = 34, 37%), principal investigators (*n* = 24, 26%), trial managers (*n* = 18, 19%), researchers involved in frontline recruitment (*n* = 10, 11%), trial methodologists (*n* = 4, 4%) and other (*n* = 3, 3%). Respondent’s experience in neonatal trial recruitment was < two years’ (*n* = 13), 2–6 years (*n* = 23), 7–10 years (*n* = 24) and > 10 years’ experience (*n* = 33).

Not all responders answered every question. Of 78 respondents who responded to item 12 which sought information on ‘*the location of the most recent neonatal trial you were involved in recruiting to’,* 74% indicated the UK, 26% the Republic of Ireland and 9% elsewhere. A high proportion of respondents (87%) agreed that it would be helpful to receive training and education about neonatal trial recruitment, even though most (83%) had previously received such training. Of those that had previously received training, 32% indicated that they had received training specific to neonatal trials, and 64% had received training about trials in general.

**Fig. 2:**
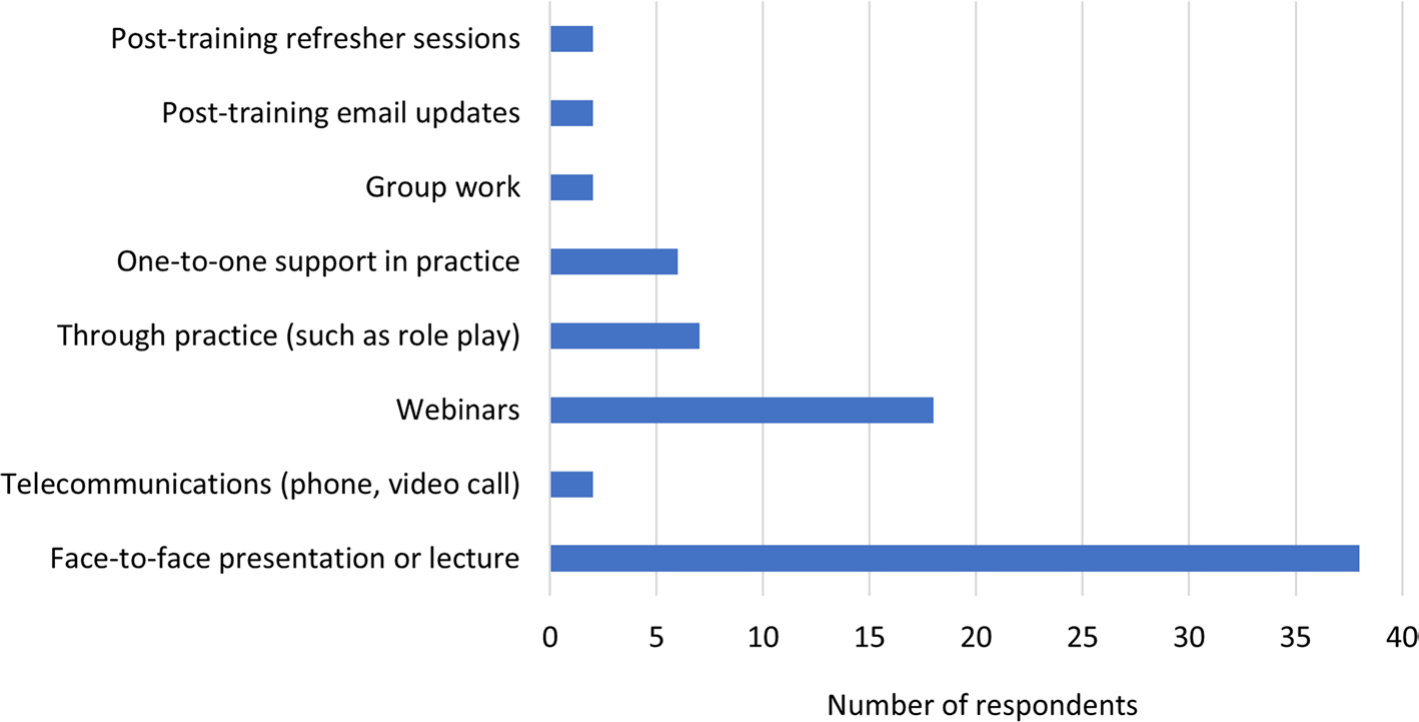
Preferred method of education and training delivery

**Fig. 3:**
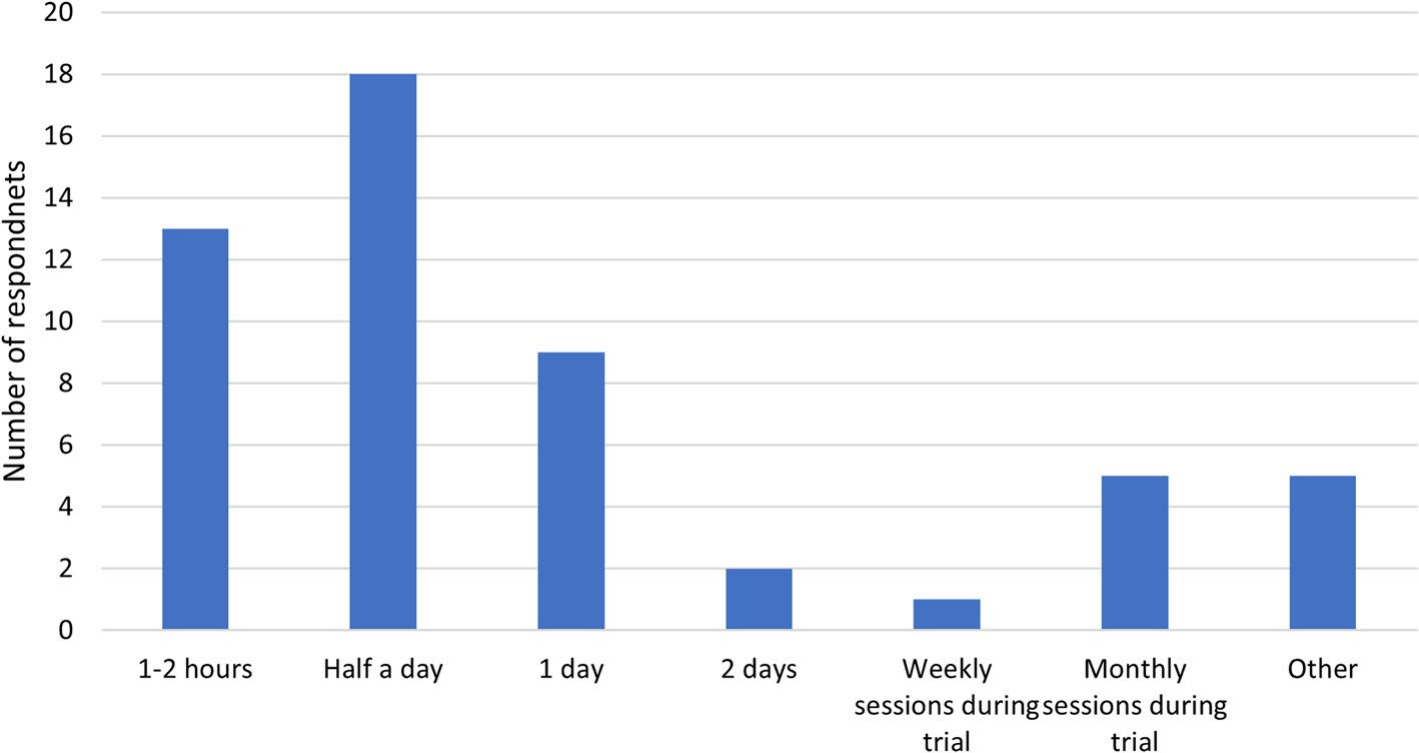
Duration of training sessions

Respondents were asked to rank a list of eight training delivery methods in order of preference. The preferred method was a face-to-face presentation or lecture format, followed by webinars, one-to-one support in practice, and through practice such as roleplay (Fig. 2). Post-training refresher sessions were ranked low by participants, however, one respondent commented that these sessions should be provided regardless of the method of training originally provided. Respondents held mixed views as to the optimal duration of training sessions (Fig. 3).

Regarding supportive education and training materials, practical checklists and top tips documents were the most popular, followed by lecture notes/slides and template recruitment materials. Reading lists or reading material was the least preferred option.

A list of sixteen trial recruitment topics was provided to respondents who were asked to rate these on a five-point scale from 1 = extremely beneficial to 5 = not at all beneficial. The top three topics in the ‘extremely beneficial’ category included background information on the study (65%), informed consent (65%), and participant eligibility (56%) (Table 1).

**Table 1:** Aspects of trials that would be beneficial to have training on (data from phase-2 survey)

Aspect^a^	1	2	3	4	5
Background information on the study	47 (65%)	24 (33%)	1 (1%)	0 (0%)	0 (0%)
Informed consent	47 (65%)	24 (33%)	0 (0%)	1 (1%)	0 (0%)
Participant eligibility	40 (56%)	27 (38%)	5 (7%)	0 (0%)	0 (0%)
Participants' needs receiving information	38 (53%)	29 (40%)	5 (7%)	0 (0%)	0 (0%)
Information specific to the trial topic area	37 (51%)	35 (49%)	0 (0%)	0 (0%)	0 (0%)
Recruitment challenges	35 (49%)	32 (45%)	4 (6%)	0 (0%)	0 (0%)
Recruitment pathways	34 (48%)	32 (45%)	5 (7%)	0 (0%)	0 (0%)
Recruitment materials	33 (46%)	35 (49%)	4 (6%)	0 (0%)	0 (0%)
Randomisation	32 (45%)	34 (48%)	3 (4%)	2 (3%)	0 (0%)
Completing trial documentation	32 (44%)	29 (40%)	10 (14%)	1 (1%)	0 (0%)
Participants' treatment options	31 (44%)	32 (45%)	8 (11%)	0 (0%)	0 (0%)
Equipoise	31 (43%)	34 (47%)	5 (7%)	2 (3%)	0 (0%)
Blinding	26 (36%)	34 (47%)	9 (13%)	2 (3%)	1 (1%)
Bio samples	25 (35%)	30 (42%)	9 (13%)	6 (8%)	2 (3%)
General information on trials	22 (31%)	40 (57%)	3 (4%)	4 (6%)	1 (1%)
Management of the trial team	22 (31%)	32 (46%)	11 (16%)	5 (7%)	0 (0%)

1: Extremely beneficial; 2: Beneficial; 3: Unsure; 4: Not beneficial; 5: Not at all beneficial

^a^Note: not all respondents answered every question

Respondents also had an option of adding any other aspects of trial recruitment that they thought would be beneficial for training. Fifteen participants responded to this item with topic areas including communication skills (building rapport with parents, approaching distressed parents, building empathy), PPI in trial design and training design/delivery, trial monitoring, and embedding trials as a research culture in a unit. These responses also reflect free-text comments provided by respondents in relation to the barriers and facilitators to trial recruitment, which were coded and organised into seven representative categories (Table 2).

**Table 2:** Perspectives on barriers and facilitators to recruitment (based on data from phase-2 survey)

Category	Key perspectives and recommendations (*n* = number of respondents contributing to the recommendation)
Trial design	- Public and patient involvement in the trial design is important so that participants' and staff needs are considered (*n* = 5) - Practically feasible trial processes and research questions are important (*n* = 4)
Training	- Sufficient training, education, and written guidance for those responsible for recruitment is critical for trial recruitment (*n* = 18)
Staff buy-in	- Improved awareness of trials (*n* = 13) amongst staff and encouraging a research culture and ‘buy in’ of staff members through building motivation, enthusiasm (*n* = 20) and providing clear information about the trial is needed (*n* = 13)
Research culture and knowledge	- The benefit of approaching parents early (at antenatal stage if possible) was highlighted so that parents are made aware early of clinical trials and in providing time to consider the trial or be advised that they may be approached to take part in a trial (*n* = 4) - Building participant trust in the research process is important for successful recruitment (*n* = 10)
Staff communication skills and rapport with potential participants	- Appropriate communication skills of staff (including the timing of when to approach parents), considering the often sensitive and distressing context of neonatal trials, are necessary (*n* = 13) - Due to the nature of many neonatal trials, the recruitment time is narrow and often in the immediate post-birth period, creating challenges for recruiters (*n* = 14) - Parental fear and uncertainty amongst parents about the potentially harmful effects on their baby during a time that is already distressing can present as a barrier to recruitment (*n* = 11); being able to build a rapport is required (*n* = 6)
Team support and dedicated time	- A dedicated research nurse and active engagement from the clinical team and PI, and ensuring multiple staff members are trained in recruitment and consent specific to the trial are important (*n* = 35) - Limited staff availability, a lack of dedicated time, and competing with other trials are barriers that require consideration (*n* = 3)
Participant documentation	- Clear documentation for potential trial participants is important (*n* = 11)

### TCIDC workshops

During Workshop 1, the TCIDC shared their feedback and experiences and new ideas and recommendations for TRAIN. The TCIDC participants emphasised the importance of building a rapport and communicating empathetically with parents when inviting their infant to take part in a trial, and that this element should feature as a significant component of the TRAIN intervention. During workshop 1 there was considerable discussion around developing tools to help recruiters see the recruitment scenario from a parent’s perspective, which was deemed challenging given their busy caseloads. Summary recommendations arising from workshop 1 are provided in Table 3, with additional detail provided in Additional file 3.

**Table 3:** Summary recommendations from Workshop 1 (based on workshop participant’s input)

TCIDC recommendation	Details
A set of slides summarising the protocol	Knowing the protocol well allows the recruiter space to focus on building trust and rapport with parents
A graphic summarising the protocol	A resource for recruiters to easily refer to
A session to consider challenging Qs	Questions that may arise from parents that are not included in the protocol
‘Pause and Think’ message	To remind recruiters to take a moment before approaching parents, consider the parents' perspective and the wider context of the scenario for them
A lanyard as a wearable reminder	With a summary of the trial protocol and a reminder to ‘pause and think’
A set of slides outlining the key points to consider when deciding if it’s the right time to approach parents	Timing, the importance of the study, honesty, what else is going on in the ward, and check-in with the parents
A video message from parents	Sharing their experience of trial recruitment to help recruiters understand the parent’s perspective
A role play exercise	Inviting recruiters to role-play recruitment scenarios and take on the role of both the parent and the recruiter. And giving the recruiters permission to accept that it is a difficult task to recruit for neonatal trials
An example script of a recruitment conversation	The steps and order of example recruitment conversations, as a means of building rapport

The research team analysed the discussion transcripts from Workshop 1 and collated these in an overview table. This helped map the proposed changes and new ideas to the existing draft intervention and further helped refine and develop the intervention. The updated draft intervention was circulated to the TCIDC before Workshop 2. During the second workshop, the TCIDC provided their final feedback and recommendations. Recommendations from Workshop 2 included points mainly related to the intervention resources' order, structure, and format. Following workshop 2 TRAIN was finalised and prepared by the research team for acceptability testing.

### TRAIN

The resulting intervention has five primary learning outcomes, such that, by the end of the intervention recruiters attending the education and training session will:

1. Understand the trial protocol and be able to explain to parents what taking part will involve
2. Understand and be able to explain the process of randomisation to parents
3. Be aware of factors to consider when approaching parents for recruitment of their neonate to a trial
4. Understand and be cognisant of parents’ perspectives when recruiting their neonates to a trial
5. Be prepared to engage in a recruitment conversation

TRAIN is designed with the intention that the training is offered to recruiters before trial recruitment begins, although the Units (Table 4) can be provided throughout trial recruitment as refresher sessions as necessary. TRAIN can be delivered online or in-person/face-to-face. A detailed trainer manual describing specifically how TRAIN should be delivered was compiled alongside the intervention resources. Once evaluated for effectiveness in a future definitive intervention trial, and finalised, the intention is that a representative from any trial team can follow TRAIN’s manual guidance in providing the education and training independently. TRAIN’s three core learning units are:

- Unit 1: The trial protocol (50 min)
- Unit 2: Understanding randomisation (5 min)
- Unit 3: Approaching and engaging with parents (70 min)

**Table 4:** Overview of the TRAIN intervention and resources

Unit	Content	Resources
Unit 1: The trial protocol (50 min)		
1.1 Introduction (10 min)	- Welcome & Introduction	1.1 Introduction (presentation slides)
1.2 The trial protocol (15 min)	- Aim/importance of the trial - Eligibility criteria - What taking part will involve including potential harms and benefits of the study	1.2 Trial Protocol (presentation)
1.3 Recruitment pathway (10 min)	- An exercise asking participants to map out the host trial pathway to assess their understanding of the information from 1.2 Trial Protocol	1.3 Recruitment Pathway exercise 1.3 Infographic (diagram summarising the protocol)
1.4 Challenging questions (10 min)	- Discussion on issues/challenging questions parents may have beyond the protocol information and how one might address these	1.4 Challenging Questions
1.5 Close (5 min)	- Questions/comments	
Unit 2: Understanding randomisation (5 min)		
2.1 Randomisation (5 min)	- A video explaining the process of randomisation to assist recruiters in explaining the process to parents of neonates who are being invited to take part in a trial	2.1 Randomisation Video
Unit 3 Approaching and engaging with parents (70 min)		
3.1 Approaching parents (30 min)	- Critical considerations for recruiters before approaching parents about the possibility of their neonate being involved in a trial	3.1 Approaching parents 3.1 Infographic 3.1 Parent video vignettes 3.1 Lanyard
3.2 Engaging with parents^a^ (15 min)	- A template recruitment conversation and order of topics with examples of opening sentences	3.2 Engaging with parents 3.2 Recruitment conversation guide
3.3 Practicing recruitment (20 min)	- Roleplay session to work through challenging recruitment scenarios, with examples specific to neonatal trials. With feedback	3.3 Practicing recruitment
3.4 Close (5 min)	- Final questions/comments	

^a^The Qualitative Research Integrated within Trials (QuinteT) team of researchers at Bristol University, of whom co-author NM is a member, pioneer approaches to optimise recruitment and informed consent to randomised controlled trials (https://www.bristol.ac.uk/population-health-sciences/research/groups/social-sciences-health/quintet/). In unit 3.2 we adapted some of QuinteT’s findings (see, for example, https://doi.org/10.1016/j.jclinepi.2008.02.010, https://doi.org/10.1186/1745-6215-15-5, https://doi.org/10.1016/j.jclinepi.2016.02.002, and https://doi.org/10.1186/s13063-017-2048-7) to the context of neonatal trials to consider how we engage with parents about their infants taking part in a trial

Unit 1 is trial-specific and focuses on the trial protocol. Units 2 and 3 are generic with applicability to any neonatal trial. Table 4 presents an overview of each unit and the related resources.

### TRAIN acceptability assessment

A total of 11 recruiters to neonatal trials registered to attend an acceptability training session. These 11 were distinct from those who participated in the phase 2 survey as the invite for the training sessions was extended globally. Of the 11 who initially registered, seven did not attend. Thus, only four recruiters took part, two in each of the two training workshops. These recruiters were from Italy (*n* = 2), Ireland (*n* = 1), and the United States (*n* = 1).

Feedback on TRAIN was positive. All four participants commented on the value of the training Units and resources, especially the ‘recruiter lanyard’ resource in Unit 1 and the parent video vignettes in Unit 3. Participants commented that if there was more flexibility in how the training is facilitated (e.g., training divided across workshops of shorter duration, rather than in one workshop), this would likely make it more accessible and enabling for recruiters to attend. The current set-up of two hours was also challenging given the depth of material being covered, and the attention required to assimilate the information; thus, it was suggested that the Units should be delivered in two sessions; Unit 1 on its own and then Units 2 and 3 together.

The recruiters suggested that Units 2 and 3 would be beneficial to incorporate into Good Clinical Practice training or as part of induction training for new staff joining neonatal units. Participants also commented that the role play session in Unit 3 was only appropriate if all TRAIN participants were recruiting to the same trial. One also noted that facilitating this session would require a particular set of facilitation skills that could not be learned from guidance in the training manual alone. One other suggestion from the participants included translating the training and resources to multiple languages to improve its accessibility.

The baseline survey, completed by all 11 who registered for a workshop, indicated that the clinical setting or trial site was active or extremely active in recruiting neonates and their parents to participate in neonatal trials for 72%. When asked about the level of support provided for the recruiters in their clinical setting/trial site, 64% reported setting/trial sites as supportive or extremely supportive, 27% were not supportive/not at all supportive, and 9% were unsure. When asked about recruiting participants to a neonatal trial, 50% of respondents felt prepared or extremely prepared, 42% were unsure, and 8% felt not at all prepared. Regarding recruiter confidence and knowledge, 75% felt confident or extremely confident, 17% were unsure, 8% were not confident, 67% felt knowledgeable or extremely knowledgeable, and 25% were unsure.

All four recruiters who took part in TRAIN completed the follow-up survey, and all four responded that following the training they felt prepared, confident or extremely confident, and knowledgeable or extremely knowledgeable. When asked to rate each of the elements of the TRAIN intervention on how useful to neonatal trial recruitment they perceived them to be, one was unsure about the duration of the training, and all four rated the remaining elements as extremely useful or valuable.

## Discussion

Poor recruitment has negative consequences and trial extensions are costly. When recruitment targets are not met, research questions are left unanswered, wasting money and participants’ time. TRAIN, underpinned by evidence syntheses and an engaged Partnership approach to intervention development, offers a comprehensive training and education intervention for recruiters to neonatal trials. The package is unique in that the views of recruiters on their education and training needs as well as the voices of those being recruited critically informed the intervention. Although recruiter training activities exist in various forms [17] evidence for the effectiveness of recruiter education and training is limited [14] and no studies were identified that focused specifically on training recruiters to neonatal trials. Furthermore, while several other recruiter training interventions, albeit in other healthcare areas, were designed for recruitment to a specific host trial [18–20], TRAIN offers both trial specific (Unit 1) and trial generic training (Units 2 and 3). This will be important for reducing trial team burden with regards to recruiter training resource development in future neonatal trials. Furthermore, TRAIN was developed such that the Units can be offered prior to trial commencement, implemented as a refresher, or implemented in response to recruitment issues during the trial, thus offering considerable flexibility to trial teams in how they might use TRAIN. This also distinguishes TRAIN from other training interventions, with several implemented in response to recruitment issues in an ongoing trial only [21–24] or embedded from the start of the trial [25–28].

A considerable portion of TRAIN (Unit 3) focuses exclusively on approaching and engaging with parents of neonates about the trial. Communicating information about a trial is important for any trial participant. This, however, has further significance in the context of neonatal trials. For example, Duley and colleagues [15] report that parents often declined to take part in perinatal trials due to burden, inconvenience, or worries about risks to their baby. Parents wish to feel informed yet report that the time provided to decide was insufficient, especially as many also wanted input from others before making their decision. Critically, Duley and colleagues described how parents reported that being approached to take part in a trial compounded their stress and worries, especially if they were approached at an inappropriate time [15]. Parent contributors in the TCIDC reinforced these findings and emphasised the importance of recruiters building a rapport with parents prior to initiating trial recruitment discussions, as well as stopping to pause and consider whether the timing of the approach was appropriate.

When recruiting to neonatal trials, recruiters also need to be cognisant of the maternal parent having recently given birth. For example, a qualitative study that explored the experiences and views of women (*n* = 22) and healthcare professionals (*n* = 27) of recruitment to a trial [29], found that while clinical staff placed emphasis on imparting information in clear and succinct ways, the reality of their post-birth situation for many women led them to make quick decisions without full engagement or an understanding of the potential risks of trial participation. For this reason, women suggested that information about a neonatal trial should be provided in the antenatal period to help ensure that consent to take part is informed [29]. Arguably, this approach could produce opposing effects, such as causing undue concern or worry, especially as many women will not be approached if the trial is focused exclusively on neonates who are unwell.

### Strengths and limitations

The comprehensive development of TRAIN is a particular strength in this study, with all involved stakeholders contributing to the final version of TRAIN. Although a considerable depth of data was provided by 93 respondents to the survey, we are unclear if this number reflects a sufficient sample size to affect representation as the precise target population number is unknown. Importantly, however, the respondents to the survey represented all categories of recruiters (i.e., front-line clinicians, trials managers, etc,.) and with a range of years of experience in neonatal trial recruitment. Securing recruiters for TRAIN’s acceptability assessments, however, was challenging, with four of 11 recruiters only taking part in the assessments. Nonetheless, the recruiters that did take part provided valuable insight into the acceptability of TRAIN, and provided meaningful recommendations for refinement, for example, having greater flexibility in the format for delivering TRAIN and facilitating training Units across two rather than one session. It would have been beneficial, however, to directly match individual pre- and post-training survey results, however, for reasons of confidentiality and anonymity, only aggregated results are available. The accrual of seven recruiters to the acceptability assessments highlighted difficulties that can exist with recruiter training engagement, especially when many recruiters will also hold busy clinical roles. Engagement of recruiters in trial training workshops has been identified in previous research and suggestions to mitigate low engagement discussed (e.g., personal engagement, encouragement by the study chief investigator to attend, and a long lead-in time for training) discussed [29]. These issues will require consideration in any future TRAIN scale-up, piloting, and effectiveness testing.

## Conclusion

TRAIN offers a comprehensive co-produced training and education intervention for recruiters to neonatal trials. TRAIN was deemed acceptable, with minor modification, by neonatal trial recruiters. Scale-up of TRAIN with formal pilot testing and testing for effectiveness in a large cluster randomised trial is required. Future intentions for this would be to embed TRAIN as a Study Within A Trial (SWAT) in a future pan-European or International cluster trial whereby half of the recruiting sites involved would receive TRAIN and the others standard training or trial training as usual for the site. To support scale-up, however, funding will be required; thus, grant calls that specifically support comparative intervention or SWAT research will be targeted.

### Supplementary Information

**Additional file 1.** TRAIN Survey (final).

**Additional file 2.** Pre and post training surveys.

**Additional file 3.** Recommendations from TCIDC Workshop 1.

## Data Availability

Publicly accessible data are available in the manuscript or in the Additional files. The intervention resources and the training manual are unavailable until large scale effectiveness testing is complete. The corresponding author can be contacted for additional information if required.

## Abbreviations

CTUs: Clinical Trial Units
HRB-TMRN: Health Research Board-Trials Methodology Research Network
HTA: Health Technology Assessment
INFANT: Irish Centre for Maternal and Child Health Research
JLA-PSP: James Lind Alliance-Priority Setting Partnership
MRC: Medical Research Council
NPEU: National Perinatal Epidemiology Unit
PPI: Public and Patient Involvement
TCIDC: TRAIN Cooperative Intervention Development Committee
TRAIN: Training tRial recruiters, An educational INtervention
UK: United Kingdom

## References

[1] Treweek S, Pitkethly M, Cook J, Fraser C, Mitchell E, Sullivan F (2018). Strategies to improve recruitment to randomised trials. Cochrane Database Syst Rev.

[2] Watson JM, Torgerson DJ. Increasing recruitment to randomised trials: a review of randomised controlled trials. BMC Med Res Methodol. 2006; 34. 10.1186/1471-2288-6-34. 10.1186/1471-2288-6-34 PMC1559709 16854229

[3] Sully BGO, Julious SA, Nicholl J (2013). A reinvestigation of recruitment to randomised, controlled, multicenter trials: a review of trials funded by two UK funding agencies. Trials.

[4] Kitterman DR, Cheng SK, Dilts SM, Orwoll ES (2011). The prevalence and economic impact of low-enrolling clinical studies at an academic medical center. Acad Med.

[5] Smith V, Clarke M, Begley C, Devane D (2015). SWAT-1: The effectiveness of a 'site visit' intervention on recruitment rates in a multicentre randomised trial. Trials.

[6] Fletcher B, Gheorghe A, Moore D, Wilson S, Damery S (2012). Improving the recruitment activity of clinicians in randomised controlled trials: a systematic review. BMJ Open.

[7] Monaghan H, Richens A, Colman S, Currie R, Girgis S, Jayne K (2007). A randomised trial of the effects of an additional communication strategy on recruitment into a large-scale, multi-centre trial. Contemp Clin Trials.

[8] DasMahapatra P, Raja P, Glibert J, Wicks P (2017). Clinical trials from the patient perspective: survey in an online patient community. BMC Health Serv Res.

[9] Bull J, Uhlenbrauck G, Mahon E, Fulong P, Roberts J. Barriers to clinical trial recruitment and possible solutions: A stakeholder survey. App Clin Trials. 2015. Accessed online 14 Jul 2022 via https://www.appliedclinicaltrialsonline.com/view/barriers-clinical-trial-recruitment-and-possible-solutions-stakeholder-survey.

[10] Crocker JC, Farrar N, Cook JA, Treweek S, Woolfall K, Chant A, Bostock J, Locock L, Rees S, Olszowski S, Bulbulia R (2020). Recruitment and retention of participants in UK surgical trials: survey of key issues reported by trial staff. BJS Open.

[11] Healy P, Galvin S, Williamson PR, Treweek S, Whiting C, Maeso B (2018). Identifying trial recruitment uncertainties using a James Lind Alliance Priority Setting Partnership – the PRioRiTy (Prioritising Recruitment in Randomised Trials) study. Trials.

[12] Bower P, Brueton V, Gamble C, Treweek S, Smith CT, Young B (2014). Interventions to improve recruitment and retention in clinical trials: a survey and workshop to assess current practice and future priorities. Trials.

[13] Fallowfield L, Langridge C, Jenkins V (2014). Communication skills training for breast cancer teams talking about trials. Breast.

[14] Delaney H, Devane D, Hunter A, Hennessy M, Parker A, Murphy L (2019). Limited evidence exists on the effectiveness of education and training interventions on trial recruitment: a systematic review. J Clin Epidemiol.

[15] Duley L, Dorling J, Ayers S, Oliver S, Yoxall CW, Weeks A, et al. Improving quality of care and outcome at very preterm birth: the Preterm Birth research programme, including the Cord pilot RCT. Programme Grants Appl Res. 2019;7(8):1–316. 31566938

[16] Delaney H, Devane D, Hunter A, Treweek S, Mills N, Gamble C, et al. A concept analysis of ‘trial recruitment’ using the hybrid model [version 2; peer review: 2 approved]. HRB Open Res. 2022; 3:92. 10.12688/hrbopenres.13173.2. 10.12688/hrbopenres.13173.1 PMC9021666 35510227

[17] Delaney H, Hunter A, Devane D. Smith V. Education and training interventions for recruiters to trials in healthcare; A Qualitative Content Analysis. Trinity Health and Education International Research Conference (THEconf2020). Ireland: Trinity College Dublin; 2020. https://nursing-midwifery.tcd.ie/events-conferences/THEconference/THEconference2020/index.php.

[18] Kimmick CG, Peterson BL, Kornblith AB, Mandelblatt J, Johnson JL, Wheeler J (2005). Improving accrual of older persons to cancer treatment trials: A randomized trial comparing an educational intervention with standard information: CALGB 360001. J Clin Oncol.

[19] Tilley B, Mainous A, Smith D, McKee M, Amorrortu R, Alvidrez J (2017). Design of a cluster-randomized minority recruitment trial: RECRUIT. Clin Trials.

[20] Kenyon S, Rhodes A, Taylor D (2005). A recipe for successful recruitment to a randomised controlled trial. MIDIRS Midwifery Digest.

[21] Kendall B, Städeli R, Schegg B, Olbrich M, Chen E, Harmelin-Kadouri R (2012). Clinical trial educator program - a novel approach to accelerate enrolment in a phase III International Acute Coronary Syndrome Trial. Clin Trials.

[22] Fisher L, Hessler D, Naranjo D, Polonsky W (2012). AASAP: A program to increase recruitment and retention in clinical trials. Patient Educ Couns..

[23] Maxwell AE, Parker RA, Drever J, Rudd A, Dennis MS, Weir CJ (2017). Promoting Recruitment using Information Management Efficiently (PRIME): a stepped-wedge, cluster randomised trial of a complex recruitment intervention embedded within the REstart or Stop Antithrombotics Randomised Trial. Trials.

[24] Mann C, Delgado D, Horwood J (2014). Evaluation of internal peer-review to train nurses recruiting to a randomized controlled trial–Internal Peer-review for Recruitment Training in Trials (InterPReTiT). J Adv Nurs.

[25] Goff SL, Youssef Y, Pekow PS, White KO, Guhn-Knight H, Lagu T (2016). Successful strategies for practice-based recruitment of racial and ethnic minority pregnant women in a randomized controlled trial: The IDEAS for a healthy baby study. J Racial Ethnic Health Disparities.

[26] Elliott D, Hamdy FC, Leslie TA, Rosario D, Dudderidge T, Hindley R (2018). Overcoming difficulties with equipoise to enable recruitment to a randomised controlled trial of partial ablation vs radical prostatectomy for unilateral localised prostate cancer. BJU Int.

[27] Blazeby JM, Strong S, Donovan JL, Wilson C, Hollingworth W, Crosby T (2014). Feasibility RCT of definitive chemoradiotherapy or chemotherapy and surgery for oesophageal squamous cell cancer. Br J Cancer.

[28] Lawton J, Snowdon C, Morrow S, Norman JE, Denison FC, Hallowell N (2016). Recruiting and consenting into a peripartum trial in an emergency setting: a qualitative study of the experiences and views of women and healthcare professionals. Trials.

[29] Parker A, Arundel C, Mills N, Rooshenas L, Jepson M, Donovan JL (2022). Staff training to improve participant recruitment into surgical randomised controlled trials: a feasibility study within a trial (SWAT) across four host trials simultaneously. Res Methods Med Health Sci.

