# Supplementary material for: The development and acceptability of an educational and training intervention for recruiters to neonatal trials: the TRAIN project
Source: BMC Med Res Methodol. 2023 Nov 11;23:265. doi: 10.1186/s12874-023-02086-1 (PMC10638723; doi:10.1186/s12874-023-02086-1)
Supplement: Supplementary file 1 — Additional file 1. TRAIN Survey (final). [file 12874_2023_2086_MOESM1_ESM.docx]

**Part 1**

**1. Which one of the following best describes your main role in recruitment to neonatal trials?**

While you may identify with more than one role, please tick the most appropriate current/most recent role**.**

- Researcher involved in frontline recruitment to trials (e.g., research assistant, research associate, research nurse/midwife, etc.)
- Clinician involved in frontline recruitment to trials (e.g., doctor, nurse, midwife, other allied health care professional)
- Principal investigator
- Trial manager and co-ordinator/clinical research co-ordinator
- Trial methodologist (someone who specializes in methods of how trials are designed, including recruitment processes)
- Other (please specify)

**2. What do you think is the main facilitator, if any, for optimising recruitment in neonatal trials? (Please use text box below)**

Text box

**3. What do you think is the main barrier, if any, to optimising recruitment in neonatal trials? (Please use text box below)**

Text box

**4. Have you ever received structured, formal training and education about trial recruitment? (e.g., a training course/workshop of any duration, in any format; this could include, for example, standardised training in taking consent, structured training on recruiting to a trial during a site initiation visit or Good Clinical Practice training)**

Yes

No [route to Q6]

**5a.** **Was the training you received specific to… (Please select all that apply)**

Neonatal trials

Trials in general

Trials in another specific healthcare field/condition (please specify) ____________

**5b. Please describe what was involved in the training and education in the text box below (this might include the duration, format (online/face-to-face), whether it was specific to aspects of recruitment to trials/a trial, etc.)**

Text box

**6. Irrespective of whether you ever received training and education about trial recruitment, do you think it would be helpful for you to receive training and education about recruitment to neonatal trials?**

Yes

No [route to Part 2]

**7a. Please indicate from the list below which trial aspects you think it would be beneficial to have training and education on, to help recruitment to neonatal trials…(please select one option on the scale from extremely beneficial to not at all beneficial)**

|  | Extremely beneficial | Beneficial | Unsure | Not beneficial | Not at all beneficial |
| --- | --- | --- | --- | --- | --- |
| General information on randomised control trials |  |  |  |  |  |
| Background information on the study |  |  |  |  |  |
| Information specific to the trial topic area |  |  |  |  |  |
| Recruitment challenges |  |  |  |  |  |
| Recruitment pathways |  |  |  |  |  |
| Recruitment materials (e.g., invitation letters and patient information leaflets) |  |  |  |  |  |
| Management of the trial team |  |  |  |  |  |
| Equipoise |  |  |  |  |  |
| Potential trial participants’ treatment options and preferences |  |  |  |  |  |
| Potential trial participants’ needs when receiving information about the trial |  |  |  |  |  |
| Randomisation |  |  |  |  |  |
| Informed consent |  |  |  |  |  |
| Completing trial documentation (e.g., case report forms/e-case report forms/serious adverse event forms/data collection forms) |  |  |  |  |  |
| Bio samples in trials (e.g., blood or tissue) |  |  |  |  |  |
| Participant eligibility |  |  |  |  |  |
| Blinding |  |  |  |  |  |

**7b. If there are any other aspects of the recruitment process that would benefit from training and education, please describe in the text box below…**

Text box

**Part 2. Some questions about your recruitment training and education preferences**

If you were to take part in training and education for trial recruitment…

**8. What would be your preferred method of delivery? Please list the options in order of preference from most preferred as 1 to least preferred as 8**

| Face-to-face presentation or lecture |  |
| --- | --- |
| Telecommunications (phone, video call) |  |
| Webinars |  |
| Through practice (such as role play) |  |
| One-to-one support in practice |  |
| Group work |  |
| Post-training email updates |  |
| Post-training refresher sessions |  |

**If you have any other preferred methods of delivery, please specify below**

**Text box**

**9. What type of supportive education and training materials would you prefer to receive?**

**Please list the options in order of preference from most preferred as 1 to least preferred as 5**

| Reading list and/or specific documents to read in advance of training |  |
| --- | --- |
| Practical checklist |  |
| Top tips document |  |
| Lecture notes (including a copy of the teaching slides/presentation) |  |
| Template recruitment materials (such as invitation letters and patient information leaflets) |  |

**If there are any other supportive education and training materials, you would prefer to receive please specify below**

**Text box**

**10. How long do you think the training should be? (please select one option)**

1-2 hours

Half a day

1 day

1.5 days

2 days

Weekly sessions for duration of trial

Monthly sessions for duration of trial

A mix of the above

Other (please specify)

**Part 3. Some questions about you**

To make sure we gather responses from a wide range of people, it would be helpful to know a little more about you. Your answers will be treated in confidence and no individual will be identified when the results are presented.

**11. How many years have you been involved in recruitment to neonatal trials? (Please select one option)**

Less than 2 years

2-6 years

7-10 years

Greater than 10 years

**12. What was the main location of the most recent neonatal trial you were involved in recruiting to? (Please select one option)**

England

Scotland

Wales

Northern Ireland

Republic of Ireland

Other (please specify)

**13. Gender? (Please use text box below or click Next if you would prefer not to answer)**

**___________________**

**14. What was the subject area of the most recent neonatal trial you were involved in recruiting to? (e.g., preterm birth, neonatal feeding etc.)**

Text box

**15. Next steps after this survey**

Once this survey has closed, we will be asking some people to take part in an online focus group or one-to-one discussion about how an education and training intervention might enhance neonatal trial recruitment processes. Would you consider taking part in an online focus group or one-to-one discussion?

Yes

No [route to Thank You page]

**16.** Thank you for considering taking part further in this study. Please provide details for your preferred method of contact to arrange potentially taking part in an online focus group or one-to-one discussion. This will be kept confidential and secure, in accordance with the Data Protection Act (Amended) 2008 and General Data Protection Regulation (2016)

Name:

Email:

**Thank you**

Thank you for taking part in our survey, your answers are important to us and we really appreciate your time.

Your answers have now been submitted.
