# Supplementary material for: The development and acceptability of an educational and training intervention for recruiters to neonatal trials: the TRAIN project
Source: BMC Med Res Methodol. 2023 Nov 11;23:265. doi: 10.1186/s12874-023-02086-1 (PMC10638723; doi:10.1186/s12874-023-02086-1)
Supplement: Supplementary file 2 — Additional file 2. Pre and post training surveys. [file 12874_2023_2086_MOESM2_ESM.docx]

**Baseline survey**

1. **How prepared do you feel for recruiting participants to a neonatal trial?**

- Not at all prepared
- Not prepared
- Unsure
- Prepared
- Extremely prepared

1. **How confident do you feel in recruiting participants to a neonatal trial?**

- Not at all confident
- Not confident
- Unsure
- Confident
- Extremely confident

1. **How knowledgeable did you feel about recruiting participants to a neonatal trial previously?** *(Please choose not applicable if you have not recruited previously)*

- Not at all knowledgeable
- Not knowledgeable
- Unsure
- Knowledgeable
- Extremely knowledgeable
- Not applicable

1. **In your opinion, how research active is your clinical setting/trial site?**

- Not at all active
- Not active
- Unsure
- Active
- Extremely active

1. **What level of support do you think is provided to recruiters to neonatal trials in your clinical setting/trial unit?**

- Not at all supportive
- Not supportive
- Unsure
- Supportive
- Extremely supportive

1. **Please indicate the type of clinical setting where you recruit to a neonatal trial**

- Maternity
- General Hospital
- Community
- Other (please specify)

1. **If you are currently recruiting, what stage in recruitment is the trial currently at?**

- Not applicable (not recruiting)
- Month 1 of recruitment
- Month 2-4 of recruitment
- Months 5-8 of recruitment
- 8 months or more of recruitment

***End: Thank you for taking the time to complete this survey – please click ‘Done’ to submit your answers***

**Post intervention survey (1 week after training)**

1. **After taking part in the TRAIN intervention, how prepared do you feel for recruiting participants to a neonatal trial?**

- Not at all prepared
- Not prepared
- Unsure
- Prepared
- Extremely prepared

1. **After taking part in the TRAIN intervention, how confident do you feel in recruiting participants to a neonatal trial?**

- Not at all confident
- Not confident
- Unsure
- Confident
- Extremely confident

1. **After taking part in the TRAIN intervention, how knowledgeable do you feel about recruiting to a neonatal trial?**

- Not at all knowledgeable
- Not knowledgeable
- Unsure
- Knowledgeable
- Extremely knowledgeable

1. **i) Please rate the following elements of the TRAIN intervention on how useful, to neonatal trial recruitment, you perceive them to be**

|  | Extremely useful | Useful | Unsure | Not useful | Not at all useful |
| --- | --- | --- | --- | --- | --- |
| The TRAIN intervention overall |  |  |  |  |  |
| Unit 1 Overall (The Trial Protocol) |  |  |  |  |  |
| Unit 1 Resource (Protocol summary graphic) |  |  |  |  |  |
| Unit 1 Activity A (trial pathway) |  |  |  |  |  |
| Unit 1 Activity B (challenging Qs) |  |  |  |  |  |
| Unit 2 Randomisation animation |  |  |  |  |  |
| Unit 3 Overall (Approaching parents) |  |  |  |  |  |
| Unit 3 Resource (Pause + Think graphic) |  |  |  |  |  |
| Unit 3 Parent videos |  |  |  |  |  |
| Unit 3 Lanyard |  |  |  |  |  |
| Unit 3 Recruitment conversation script |  |  |  |  |  |
| Unit 3 Role play practicing recruitment |  |  |  |  |  |
| Overall duration of the training |  |  |  |  |  |
| Overall delivery format of the training |  |  |  |  |  |

**4. ii) Any additional comments?**

Text box

**5. Are there any topic areas that you think had too little information?**

Yes/No: Please specify … Text box

**6. Are there any topic areas that you think had too much information?**

Yes/No: Please specify … Text box

**7. Are there any elements of the training that you found particularly helpful?**

Yes/No: Please specify … Text box

**8. Are there any elements of the training that you found were not particularly helpful?**

Yes/No: Please specify … Text box

**9. What would be your preferred method of delivery for the training?**

- Online
- In person (once Covid restrictions are eased)
- Other (please specify)

**10. Please provide any further suggestions or feedback on how we could improve the delivery or content of the TRAIN intervention**

Text box

**11.** **Please indicate the type of clinical setting where you recruit to a neonatal trial**

- Maternity
- General Hospital
- Community
- Other (please specify)

***End: Thank you for taking the time to complete this survey – please click ‘Done’ to submit your answers***
