# Supplementary material for: The development and acceptability of an educational and training intervention for recruiters to neonatal trials: the TRAIN project
Source: BMC Med Res Methodol. 2023 Nov 11;23:265. doi: 10.1186/s12874-023-02086-1 (PMC10638723; doi:10.1186/s12874-023-02086-1)
Supplement: Supplementary file 3 — Additional file 3. Recommendations from TCIDC Workshop 1. [file 12874_2023_2086_MOESM3_ESM.docx]

**Additional File 3: Recommendations from TCIDC Workshop 1**

| Format |
| --- |
| A combination of face to face and online. Face to face needs to be interactive and with small group work. Online pre-recorded or live elements embedded. |
| Written materials with theory to back up the interactive/face to face/pre-recorded elements |
| A multi-modal format to make it as accessible as possible - to include separate stand-alone units that can be accessed depending on need and time |
| It needs to be multi-disciplinary, and the wider team should attend at least some elements of the training |
| Mandatory vs Optional - potential to have particular modules that are mandatory |
| Pre-recorded elements should be short, and the face to face (online or in-person) can be longer but should be interactive |
| Background Info |
| A simplified summary of the protocol outlining the who, how and why of the study and potential questions/issues from parents that may not be covered in the protocol |
| Highlight the negative impact on trust if a recruiter is seen to be guessing/not clear on trial information - it’s better to admit you don’t know and find out/look it up later. |
| Outlining to parents the other trials you’ve been involved in and the positive impact of those trials to build trust and interest |
| Building trust and outlining the benefits/potential harms are most important in the initial meeting- if you can initially generate trust and portray that it is an important study that is key (details on potential harms does need to be presented in detail to parent early on) |
| Info specific to the trial area |
| Combine this section with the Background Information section |
| Highlighting the importance of the study and the relevance of the research question (often recruiters will arrive at training already with their mind made up on whether or not it’s an important research question). Present the study to recruiters in a way that will bring people along that wouldn't have necessarily bought into it. |
| Present the benefits and harms maybe as a graphic. Also highlighting that if the study is not done, that is considered as a potential harm |
| Eligibility criteria |
| Presented as a checklist but needs to be available and visible in practice (e.g., on a lanyard or poster) |
| A lanyard with eligibility criteria would be helpful but this should also include a reminder to 'pause and think' about the parents’ context and to question is this a good time to approach |
| Timing/approaching parents |
| Build awareness amongst recruiters on how to prepare before approaching a parent: look at your environment, look at the people, their faces, what’s going on around, what have the parents already/just been told, their new environment, the enormity of the situation and the trauma, put yourself in their position, what's going on in the unit in general, is there another baby who is particularly unwell at the moment, that may be influencing that particular family if they have become friendly with them, being aware of everything that's going on in the environment within the unit, not just the baby they're focusing on. |
| Do some engaging activities to help the recruiters think from the parent’s perspective (e.g., think of a traumatic time they experienced), facilitating the recruiters to accept that it is a difficult task to recruit for neonatal trials |
| Identifying when to bow out and when the timing is not right and when the parent needs someone or something else at that moment (e.g., lactation specialist etc.) |
| Communication skills/rapport |
| Roleplay with actual parents if possible or actors and multiple scenarios (and time for reflection on this) |
| The recruiter being in a better headspace to pause before approaching and less stressed so better able to build rapport – this could be helped by them feeling confident in the background info about the trial |
| Provide an example of how a conversation might go – the steps; don’t launch into a discussion about the trial, ask the parent how they are first, build a rapport, be aware of the balance and the recruiter-participant dynamic vs person-person dynamic (again a video from a parent or a video of good/bad examples) |
| Explain the importance of the order of information – info and updates on the baby’s health must always come before a discussion about the trial ‘*You have to be careful about what you come across as wanting from them’* |
| Shadowing or a buddy/mentor system for inexperienced recruiters to learn in practice |
| To consider that it is not just about mothers, both parents should be considered and be clear of who needs to give consent from the outset so not to undermine the fathers when they give consent |
| Provide information from parents on what they need, what would make them feel comfortable in giving consent |
| Parents' needs |
| A reminder to consider that the parents will be struggling both emotionally *and* physically |
| A video recording of mother and father and their experience, maybe record an interview/conversation with them to hear and understand their stories. |
